# Physical activity, sedentary behavior and risk of coronary artery disease, myocardial infarction and ischemic stroke: a two-sample Mendelian randomization study

**DOI:** 10.1007/s00392-021-01846-7

**Published:** 2021-03-27

**Authors:** Martin Bahls, Michael F. Leitzmann, André Karch, Alexander Teumer, Marcus Dörr, Stephan B. Felix, Christa Meisinger, Sebastian E. Baumeister, Hansjörg Baurecht

**Affiliations:** 1 Department of Internal Medicine B, University Medicine Greifswald, 17475 Greifswald, Germany; 2 DZHK (German Centre for Cardiovascular Research), Partner Site Greifswald, Greifswald, Germany; 3 Department of Epidemiology and Preventive Medicine, University of Regensburg, Regensburg, Germany; 4 Institute of Epidemiology and Social Medicine, University of Muenster, Muenster, Germany; 5 Institute for Community Medicine, University Medicine Greifswald, Greifswald, Germany; 6 Chair of Epidemiology, LMU München, UNIKA-T Augsburg, Augsburg, Germany; 7 Independent Research Group Clinical Epidemiology, Helmholtz Zentrum Muenchen, Munich, Germany; 8 Institute of Health Services Research in Dentistry, University of Muenster, Muenster, Germany

**Keywords:** 2 sample MR, Physical activity, Myocardial infarction, Coronary Artery Disease

## Abstract

**Aims:**

Observational evidence suggests that physical activity (PA) is inversely and sedentarism positively related with cardiovascular disease risk. We performed a two-sample Mendelian randomization (MR) analysis to examine whether genetically predicted PA and sedentary behavior are related to coronary artery disease, myocardial infarction, and ischemic stroke.

**Methods and results:**

We used single nucleotide polymorphisms (SNPs) associated with self-reported moderate to vigorous PA (*n* = 17), accelerometer based PA (*n* = 7) and accelerometer fraction of accelerations > 425 milli-gravities (*n* = 7) as well as sedentary behavior (*n* = 6) in the UK Biobank as instrumental variables in a two sample MR approach to assess whether these exposures are related to coronary artery disease and myocardial infarction in the CARDIoGRAMplusC4D genome-wide association study (GWAS) or ischemic stroke in the MEGASTROKE GWAS. The study population included 42,096 cases of coronary artery disease (99,121 controls), 27,509 cases of myocardial infarction (99,121 controls), and 34,217 cases of ischemic stroke (404,630 controls). We found no associations between genetically predicted self-reported moderate to vigorous PA, accelerometer-based PA or accelerometer fraction of accelerations > 425 milli-gravities as well as sedentary behavior with coronary artery disease, myocardial infarction, and ischemic stroke.

**Conclusions:**

These results do not support a causal relationship between PA and sedentary behavior with risk of coronary artery disease, myocardial infarction, and ischemic stroke. Hence, previous observational studies may have been biased.

**Graphic abstract:**

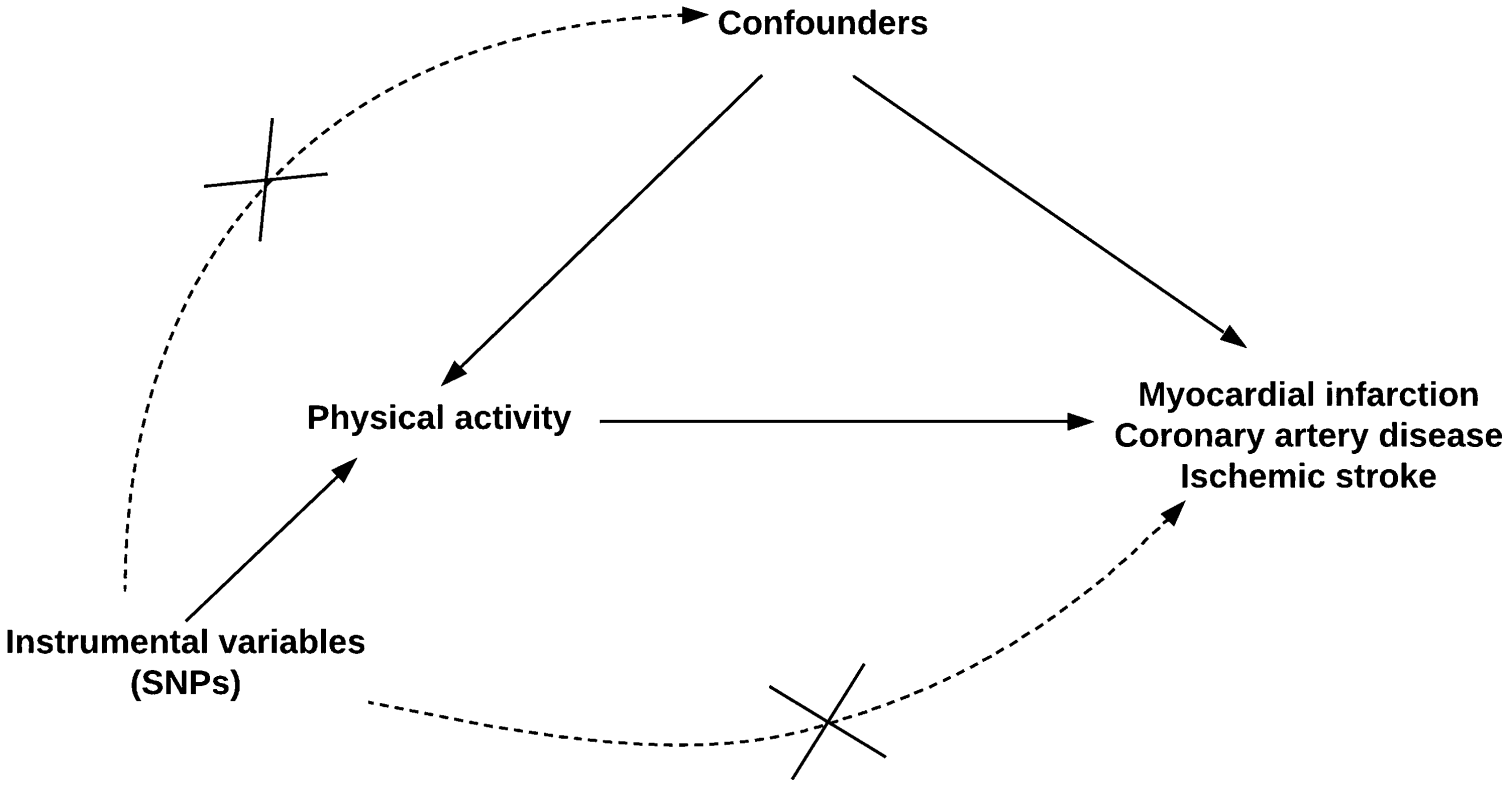

**Supplementary Information:**

The online version contains supplementary material available at 10.1007/s00392-021-01846-7.

## Introduction

Cardiovascular disease, including coronary artery disease and myocardial infarction are the main causes of morbidity and mortality worldwide [1–3]. To reduce the burden on the population and individual level of these illnesses, effective prevention measures are essential. Current guidelines endorse a physically active lifestyle to reduce the risk for cardiovascular disease [4, 5]. A recent meta-analysis included 44 studies and more than 1.5 million participants [6] and showed a clear inverse relation of moderate and intense physical activity (PA) with cardiovascular mortality. Another meta-analysis of 36 studies (including 179,393 events) reported that individuals who had an inactive lifestyle compared to those achieving the recommended 150 min of moderate PA per week had a 23% higher risk for cardiovascular mortality and 17% greater risk for incident cardiovascular disease, respectively [7]. Further, PA is recommended for the primary prevention of ischemic stroke [8]. Overall, the general consensus in the scientific community is that PA reduces the risk for coronary artery disease, myocardial infarction and ischemic stroke [5, 9, 10].

However, the causal relationship between PA and cardiovascular disease risk is not clear. One drawback of the current state of knowledge is that the majority of previous studies used questionnaires to assess PA [4]. The use of self reports may considerably influence (i.e., over-estimate) the levels of PA due to recall and social desirability biases [11, 12]. To overcome these issues, more recent studies have used device measured PA [13–18].

We tried to disentangle the role of PA and cardiovascular disease risk using a two-sample Mendelian randomization (MR). MR is a form of causal inference employing instrumental variable analysis which utilizes genetic variants (SNPs) as instruments. This method has also been referred to as nature’s randomized controlled trial, since the genetic variants are allocated randomly at conception [19, 20]. This approach allows to overcome biases of observational studies like unobserved confounding and reverse causation [21]. Two-sample MR relate exposure and outcome summary data from genome-wide association studies and with increasing sample size by forming consortia lead to increased statistical power. We performed a two-sample summary data MR analyses to explore relationships between self-reported moderate to vigorous (MVPA), accelerometer assessed PA, accelerometer-assessed fraction accelerations > 425 milli-gravities as well as sedentary behavior with risk for coronary artery disease, myocardial infarction, and ischemic stroke.

## Methods

We used a five step approach for this two-sample MR analysis. We first identified genetic variants which are associated with MVPA, accelerometer assessed PA, accelerometer assessed fraction accelerations > 425 milli-gravities as well as sedentary behavior in the UK Biobank to be used as exposures. Second, the outcome variables for this two-sample MR were identified from genome-wide association studies (GWAS) for coronary artery disease, myocardial infarction, and ischemic stroke. Third, the SNP-exposure and SNP-outcome datasets were harmonized. Fourth, we performed the two-sample MR analyses. The last step included sensitivity analyses to ensure that the assumptions for MR analyses are met.

### Instrumental variables for physical activity and sedentary behavior

Data for the genetic associations with self-reported and accelerometer-based PA to be used as exposures were obtained from two published GWAS conducted in the UK Biobank [22, 23]. The UK Biobank study is a community-based prospective cohort study conducted between 2006 and 2010 that recruited over 500,000 men and women aged 40–69 years from different socioeconomic backgrounds from 22 centers across the United Kingdom [24].

Klimentidis et al. [23] identified SNPs associated with MVPA in 377,234 UK Biobank participants using the International Physical Activity short form Questionnaire [25]. MVPA was calculated as the sum of total minutes per week of MVPA multiplied by eight, corresponding to their metabolic equivalent tasks (METs) [23]. The mean (SD) MVPA was 1,650 (2,084) MET-minutes/week. Accelerometer-based PA was measured in a subset of 103,712 participants with an Axivity AX3 triaxial accelerometer on the wrist for a seven-day-period between 2013 and 2015 [26]. After calibration, removal of gravity and sensor noise, and identification of wear/non-wear episodes the remaining 100 Hz raw triaxial acceleration data were used to calculate objective PA variables. Non-wear time was defined as consecutive stationary episodes lasting for at least 60 min where all three axes had a standard deviation of less than 13.0 milli-gravities. The values for the accelerometer-measured physical activity exposure ‘average acceleration’ was 27.9 (27.0) milli-gravities. We used two different exposures based on accelerometer-assessed PA: ‘average acceleration’ (in milli-gravities) and ‘fraction of accelerations > 425 milli-gravities’ corresponding to an intensity levels ≥ 6 METs [23]. Sedentary time was also derived from UK Biobank accelerometer data and definded as activity levels ≤ 1.5 METs [22].

### Selection of genetic instrumental variables for physical activity

We initially selected 19 SNPs associated with self-reported MVPA at a genome-wide significance level (*P* < 5 × 10^–8^) in the GWAS by Klimentidis et al. [23], using the PLINK clumping algorithm (*r*^2^ threshold = 0.001 and window size = 10 mB) [27] (Supplementary Table 1). We identified eight SNPs associated with accelerometer-measured ‘average acceleration’ at *P* < 5 × 10^–8^ [23] (Supplementary Table 2). Further, seven and six SNPs were associated with fraction accelerations > 425 milli-gravities [23] and sedentary behavior [22], respectively (Supplementary Tables 3 and 4). After the removal of SNPs exhibiting potential pleiotropic effects (see details in ‘Statistical analyses’, ‘Results’ and suppl. Table 5), 17, 7, 7 and 6 SNPs were used as instrumental variables for MVPA, accelerometer measured PA, fraction accelerations > 425 milli-gravities and sedentary behavior, respectively. UK Biobank participants were genotyped using the UK BiLEVE array and the UK Biobank axiom array.

### GWAS summary data for coronary artery disease, myocardial infarction and ischemic stroke

Genetic variants associated with coronary artery disease (42,096 cases and 99,121 controls) [28], myocardial infarction (27,509 cases and 99,212 controls), and ischemic stroke (34,217 cases and 404,630 controls) [29] were obtained from GWAS meta-analyses. These GWAS meta-analyses did not include participants of the UK Biobank but have the same ethnicity as UK Biobank participants. Coronary artery disease diagnosis was broadly defined as myocardial infarction, acute coronary syndrome, chronic stable angina or coronary stenosis of > 50% [28]. Myocardial infarction was defined based on symptoms of persistent ischemic chest pain, ischemic changes on electrocardiogram with dynamic evolution, and increases in the levels cardiac biomarkers. Stroke was defined according to the World Health Organization (WHO), i.e. rapidly developing signs of focal (or global) disturbance of cerebral function, lasting more than 24 h or leading to death with no apparent cause other than that of vascular origin. Strokes were defined as ischemic stroke based on clinical and imaging criteria. Information on participants included in each meta-analysis is provided in the appendix (suppl. Table 13 and 14).

### Standard protocol approvals, registrations, and patient consents

Written informed consent was obtained from UK Biobank study participants and ethics approval of the UK Biobank was given by the North West Multicentre Research Ethics Committee, the National Information Governance Board for Health & Social Care and the Community Health Index Advisory Group. Both GWAS studies [22, 23] were covered by the general ethical approval of the UK Biobank studies from the NHS National Research Ethics Service on 17th June 2011 (Ref 11/NW/0382).

For the studies participating in the coronary artery disease [30], myocardial infarction [30] and ischemic stroke [29] GWAS, written informed consent was obtained from study participants, legal guardian, or other proxy. Study protocols for all cohorts were reviewed and approved by the appropriate institutional review boards [29, 30]. The investigation must conform to the principles outlined in the Declaration of Helsinki.

### Statistical power

The SNPs used as instrumental variables for MVPA, accelerometer-assessed PA and fraction accelerations > 425 milli-gravities explained 0.15%, 0.24%, and 0.23% of the variance, respectively. A total of 0.22% of the variance in sedentary behavior was explained by the SNPs identified by Doherty et al. [22]. We had sufficient statistical power to detect an association between a 1 SD change in accelerometer-assessed PA and coronary artery disease (0.79), myocardial infarction (0.67), and ischemic stroke (0.85) given a type 1 error of 5% and an expected odds ratio (OR) of 0.7 (Suppl. Table 6). A priori statistical power was calculated according to Burgess [31].

## Statistical analyses

A multiplicative random effects inverse-variance weighted (IWV) estimate was used as the principal outcome for this two-sample MR [21, 32]. IVW estimates were calculated from the ratio estimates of the individual genetic variants in the IVW meta-analysis. Under the assumption of balanced pleiotropy, the random effects IVW approach provides valid causal estimates by allowing each SNP to have different mean effects compared to the fixed effects IVW [32]. For the continuous exposure variables (i.e. MVPA and accelerometer-assessed PA) the results are represented as OR per 1-SD increment in MET-minutes/week and average acceleration in milli-gravities, respectively. One SD of ‘average acceleration’ in the UK Biobank Study is approximately 8 milligravities (or 0.08 m/s^2^) of acceleration in a mean 5-s window [23]. For dichotomous exposure parameters (i.e. fraction accelerations > 425 milli-gravities and sedentary behavior) the OR for engaging in fraction acceleration ≥ 425 milli-gravities vs. fraction acceleration < 425 milli-gravities or sedentary behavior (energy expenditure ≤ 1.5 METs vs- energy expenditure > 1.5 METs) were used. All comparisons were adjusted for multiple testing using false discovery rate [33]. Analyses were performed using the TwoSampleMR (version 0.5.2) [34], MendelianRandomization (for multivariable MR analysis) and MRPRESSO (version 1.0) packages in R (version 3.6.3, Vienna, Austria)[35]. Reporting followed the STROBE-MR statement [36].

### Two-sample MR assumptions and sensitivity analyses

First, we examined the instrumental variable assumptions: i.e. the strength of the genetic instruments (IV1), IVs are not associated with confounders (IV2), and there is no residual IV-outcome association given exposure and confounders (IV3) [37]. Additional two-sample MR assumptions cannot be formally investigated but addressed by adequate choice of exposure and outcome GWAS: the causal relationship holds for both samples (2SMR1), independence of both samples (2SMR2), the variances of exposure and outcome are known (2SMR3), no measurement error in the IV-exposure association (NOME) [37, 38].

IV1 was assessed by calculating the F statistic for each instrumental variable [39]. IV2 was tested by entering each instrumental genetic variant and its proxies (*r*^2^ > 0.8) in PhenoScanner [40], GWAS catalog [41] and GWAS Atlas [42] to identify previously reported associations (*P* < 5 × 10^–8^) with confounders or cardiovascular outcomes. Previous research has identified spiroergometric markers (i.e. forced vital capacity, forced expiratory volume in 1 s, total vital capacity) [43], anthropometric markers (i.e. body fat, body mass index, hip circumference and fat free mass) [44] and education [45] as potential confounders for the relationship between physical activity and cardiovascular outcomes. The number of SNPs without potential for confounding was too low to perform a sensitivity analysis by excluding potentially confounded SNPs. Hence, we performed a multivariable MR analysis [46] to assess the relationship between genetically predicted self-reported moderate to vigorous physical activity, accelerometer-derived PA, vigorous PA and sedentary behavior on coronary artery disease, myocardial infarction and ischemic stroke adjusted for education and body mass index [47, 48]. IV3 was evaluated by performing a leave-one-out analysis to assess whether the IVW estimate was driven or biased by a single SNP.

The two-sample MR assumptions were evaluated by performing additional sensitivity analyses. The weighted median MR method is robust to potential unbalanced horizontal pleiotropy. Horizontal pleiotropy violates the exclusion restriction assumption, because in this case the instrumental variables are associated with other traits (i.e. intermediates) which influence the outcome [21]. To detect and correct for outliers of the IVW linear regression, we applied the MR-Pleiotropy RESidual Sum and Outlier (MR-PRESSO) method [34, 38]. The presence of directional pleiotropy was assessed by MR Egger regression. The intercept of this regression must be close to “zero” otherwise pleiotropy may be present [21]. We used a modified 2nd order weighting approach to estimate the Cochran’s Q statistic as a measure of heterogeneity [49].

### Data availability

Summary statistics for the physical activity GWAS by Klimentidis et al. [23] are available at https://klimentidis.lab.arizona.edu/content/data (access date: 2020/01/27) and summary data for the GWAS by Doherty et al. [22] are available at https://doi.org/10.5287/bodleian:yJp6zZmdj (access date: 2020/03/22). The GWAS for coronary artery disease and myocardial infarction [28] are available for download from the CARDIoGRAMplusC4D website (http://www.cardiogramplusc4d.org/data-downloads/). The GWAS for ischemic stroke [29] can also be downloaded on the website of the MEGASTROKE consortium (https://www.megastroke.org/download.html).

## Results

### MR analysis for MVPA, accelerometer-assessed PA, fraction accelerations > 425 milli-gravities and sedentary behaviour on coronary artery disease, myocardial infarction, and ischemic stroke

We found no evidence for an effect of genetically predicted MVPA and accelerometer-assessed PA on coronary artery disease (IVW OR per 1-SD: 1.00–1.03; *P* > 0.730), myocardial infarction (IVW OR per 1-SD: 0.99–1.16; *P* > 0.457), and ischemic stroke (IVW OR per 1-SD: 0.98–1.16; *P* > 0.244) (Tables 2 and 3). Moreover, neither fraction accelerations > 425 milli-gravities nor sedentary behavior showed a relationship with coronary artery disease (IVW OR per 1-SD: 0.77, *P* = 0.112 and OR: 0.91, *P* = 0.709), myocardial infarction (IVW OR per 1-SD: 0.75, *P* = 0.098 and OR: 0.96, *P* = 0.855), and ischemic stroke (IVW OR per 1-SD: 0.77, *P* = 0.123 and OR: 1.06, *P* = 0.748) (Tables 4 and 5).

### Two-sample MR assumptions and sensitivity analyses

To assess whether the three IVs and additional Two-Sample-MR assumptions held true, sensitivity analyses for a complete assessment of the results were performed. The strength of all genetic instruments for the exposure variables (i.e. MVPA, accelerometer-assessed PA, fraction accelerations > 425 milli-gravities and sedentary behavior) were sufficient as evidenced by the high F-statistics with values of 29.9 or higher. Hence, IV1 was not violated since weak instrument bias was not present (Table 1). To evaluate whether the IVs were not associated with confounders (IV2) the genetic variants were entered in the PhenoScanner database, the GWAS catalog and the GWAS Atlas. A large number of SNPs was related to potential confounders like education, lung function and anthropometric markers (Suppl. Table 5). Hence, a potential violation of IV2 is possible. However, we conducted multivariable MR analyses to adjust for body mass index and education. Using this approach we found similar associations compared to the univariate analyses (suppl. Tables 13–17). To ensure that there was no residual IV-outcome association given exposure and confounders (IV3) leave one out analysis were performed. These analyses yielded no significant findings for the association between MVPA, accelerometer-assessed PA and sedentary behavior with coronary artery disease, myocardial infarction and ischemic stroke (suppl. Tables 8, 9, 11). However, the leave one out analysis identified the single SNP rs34858520 (suppl. Table 10) to influence the relation of fraction accelerations > 425 milli-gravities with coronary artery disease and myocardial infarction. Further, the allele frequency of this SNP strongly differed between the exposure GWAS (EAF = 0.558) and outcome GWAS (EAF_MI_ = 0.626, EAF_CAD_ = 0.614; Table 1, suppl. Table 4).

**Table 1:** Genetic variants associated with self-reported physical activity (PA), accelerometer-assessed PA, vigorous PA and sedentary bevahior

SNP	Chr	Position (hg38)	EA	OA	EAF	beta	se	*P* value	*R*^2^	*F* statistic
Self-reported MVPA (based on a GWAS by Klimentidis et al. [23])										
rs2942127	1	204450939	G	A	0.175	0.016	0.003	3.30E−08	0.00008	30.522
rs1974771	2	54051406	G	A	0.9	-0.021	0.004	6.60E−09	0.00009	33.654
rs2114286	3	41152792	A	G	0.466	− 0.012	0.002	3.30E−08	0.00008	30.501
rs877483	3	53812714	T	C	0.433	0.012	0.002	4.00E−08	0.00008	30.132
rs2035562	3	85007370	A	G	0.328	− 0.014	0.002	3.90E−09	0.00009	34.687
rs1972763	4	158939411	C	T	0.342	0.013	0.002	3.30E−08	0.00008	30.526
rs77742115	5	18330315	T	C	0.862	− 0.018	0.003	9.60E−09	0.00009	32.922
rs2854277	6	32660307	C	T	0.917	0.032	0.005	2.60E−10	0.00011	39.959
rs1186721	7	34934990	G	A	0.684	− 0.013	0.002	4.40E−08	0.00008	29.984
rs921915	7	50188985	T	C	0.412	− 0.014	0.002	5.70E−10	0.0001	38.436
rs1043595	7	128769958	G	A	0.717	0.014	0.002	4.30E−09	0.00009	34.481
rs7804463	7	133762898	T	C	0.53	0.015	0.002	1.20E−11	0.00012	45.987
rs2988004	9	37044391	T	G	0.558	− 0.013	0.002	4.10E−09	0.00009	34.579
rs7326482	13	53463668	G	T	0.385	− 0.013	0.002	1.60E−08	0.00008	31.915
rs10145335	14	98081411	G	A	0.749	− 0.014	0.003	2.70E−08	0.00008	30.878
rs12912808	15	94748994	C	T	0.851	0.018	0.003	1.70E−08	0.00008	31.852
rs1921981	21	41050620	G	A	0.674	0.013	0.002	3.80E−08	0.00008	30.224
Accelerometer assessed average acceleration (based on a GWAS by Klimentidis et al. [23])										
rs34517439	1	77984833	C	A	0.879	0.308	0.056	4.40E−08	0.00033	29.972
rs6775319	3	18717009	A	T	0.271	0.225	0.041	3.50E−08	0.00033	30.43
rs9293503	5	88653144	T	C	0.888	0.329	0.059	2.10E−08	0.00034	31.42
rs12522261	5	152675265	G	A	0.657	0.211	0.038	3.90E−08	0.00033	30.207
rs11012732	10	21541175	A	G	0.668	0.225	0.039	5.40E−09	0.00037	34.036
rs148193266	11	104657953	A	C	0.957	− 0.51	0.092	3.10E−08	0.00034	30.67
rs59499656	18	43188344	A	T	0.656	− 0.228	0.038	2.40E−09	0.00039	35.597
Fraction accelerations > 425 milli-gravities (based on a GWAS by Klimentidis et al. [23])										
rs1856329	1	219766281	A	C	0.801	0.027	0.005	9.00E−08	0.00032	28.569
rs6433478	2	174376754	T	C	0.457	− 0.024	0.004	1.20E−08	0.00036	32.465
rs62443625	7	39013531	T	C	0.767	− 0.026	0.005	1.40E−07	0.00031	27.683
rs72633364	8	34329370	G	A	0.711	− 0.023	0.005	4.10E−07	0.00028	25.635
rs4754194	11	107219461	C	T	0.773	− 0.025	0.005	2.40E−07	0.00029	26.642
rs743580	15	74035775	A	G	0.51	0.025	0.004	1.30E−09	0.00041	36.764
rs1668835	18	24898988	T	A	0.688	− 0.023	0.004	3.10E−07	0.00029	26.184
Sedentary behaviour (based on a GWAS by Doherty et al. [22])										
rs61776614	1	2234967	C	T	0.925	0.05	0.009	3.90E−08	0.00033	30.182
rs1858242	3	68477984	A	G	0.259	0.031	0.005	3.80E−09	0.00038	34.745
rs26579	5	88689478	G	C	0.415	0.028	0.005	2.60E−09	0.00039	35.446
rs25981	5	107487207	G	C	0.531	0.028	0.005	2.70E−09	0.00039	35.366
rs6870096	5	152566250	C	G	0.321	− 0.028	0.005	2.40E−08	0.00034	31.149
rs34858520	7	72258898	A	G	0.558	0.028	0.005	4.50E−09	0.00038	34.402

*SNP* single nucleotide polymorphism, *Chr.* chromosome, *hg38* human genome assembly 38, *EA* effect allele, *OA* other allele, *EAF* effect allele frequency, *beta* beta estimate, *se* standard error, *MVPA* moderate to vigorous physical activity

A modified Q-statistic was used to identify heterogeneity across individual SNPs (suppl. Table 7). Heterogeneity was identified for the association of fraction accelerations > 425 milli-gravities (Cochran’s *Q* 13.73, *P* = 0.033) and sedentary behavior (Cochran’s *Q* 14.54, *P* = 0.013) with coronary artery disease. Additionally, significant heterogeneity was found for the relationship between sedentary behavior with myocardial infarction (Cochran’s *Q* 11.58, *P* = 0.041). The results between MVPA and ischemic stroke were also influenced by heterogeneity (Cochran’s *Q* 19.17, *P* = 0.004).

To address potential balanced horizontal pleiotropy, weighted median estimates are reported. These results were similar to the IVW estimates. MR-Presso and MR-Egger methodologies were used to assess potential pleiotropy of the genetic instruments. These results did not differ from the observed effects for MVPA, accelerometer-assessed PA, and fraction accelerations > 425 milli-gravities on coronary artery disease, myocardial infarction and ischemic stroke in the primary analyses (Tables 2, 3, 4 and 5). The MR Egger intercept test was used to find potential horizontal pleiotropy (suppl. Table 12). No horizontal pleiotropy was identified for the relation between MVPA, accelerometer-assessed PA and fraction accelerations > 425 milli-gravities with coronary artery disease, myocardial infarction, and ischemic stroke. However, directional horizontal pleiotropy was found for the association between sedentary behavior and coronary artery disease (intercept − 0.104, *P* = 0.008) as well as myocardial infarction (intercept − 0.093, *P* = 0.04).

**Table 2:** Mendelian randomization estimates between self-reported moderate-to-vigorous physical activity identified by Klimentidis et al. [23] and coronary artery disease, myocardial infarction, and ischemic stroke

Method	N SNPs	OR^a^	95% CI	*P *value
Coronary artery disease				
Inverse variance weighted	17	1.03	(0.71–1.50)	0.875
Weighted median	17	0.94	(0.57–1.54)	0.800
MR Egger	17	1.81	(0.31–10.61)	0.512
MR PRESSO	17	1.03	(0.71–1.49)	0.877
Myocardial infarction				
Inverse variance weighted	17	1.16	(0.74–1.80)	0.519
Weighted median	17	1.28	(0.74–2.23)	0.375
MR Egger	17	1.57	(0.19–12.97)	0.676
MR PRESSO	17	1.16	(0.74–1.80)	0.527
Ischemic stroke				
Inverse variance weighted	17	1.16	(0.80–1.67)	0.436
Weighted median	17	1.15	(0.71–1.87)	0.565
MR Egger	17	1.51	(0.26–8.74)	0.649
MR PRESSO	17	1.16	(0.86–1.55)	0.340

*MR PRESSO* MR Pleiotropy RESidual Sum and Outlie, *CI* confidence interval, *MET* metabolic equivalent tasks

^a^OR (odds ratio) per increase in MET/h per week for self-reported physical activity

**Table 3:** Mendelian randomization estimates between accelerometer-derived average accelerations identified by Klimentidis et al. [23] in relation to coronary artery disease, myocardial infarction, and ischemic stroke

Method	N SNPs	OR^a^	95% CI	*P* value
Coronary artery disease				
Inverse variance weighted	7	1.01	(0.96–1.06)	0.802
Weighted median	7	0.99	(0.95–1.04)	0.722
MR Egger	7	1.14	(0.94–1.38)	0.198
MR PRESSO	7	1.01	(0.96–1.06)	0.809
Myocardial infarction				
Inverse variance weighted	7	0.99	(0.95–1.04)	0.748
Weighted median	7	0.99	(0.94–1.05)	0.794
MR Egger	7	1.08	(0.88–1.32)	0.476
MR PRESSO	7	0.99	(0.95–1.04)	0.752
Ischemic stroke				
Inverse variance weighted	7	0.98	(0.92–1.05)	0.515
Weighted median	7	0.97	(0.91–1.03)	0.312
MR Egger	7	1.11	(0.84–1.46)	0.472
MR PRESSO	6	0.96	(0.90–1.02)	0.213

*MR PRESSO* MR Pleiotropy RESidual Sum and Outlier, *CI* confidence interval

^a^OR (odds ratio) per increase in milligravities for accelerometer derived physical activity

**Table 4:** Mendelian randomization estimates between fraction acceleration > 425 milli-gravities identified by Klimentidis et al. [23] in relation to coronary artery disease, myocardial infarction, and ischemic stroke

Method	N SNPs	OR^a^	95% CI	*P* value
Coronary artery disease				
Inverse variance weighted	7	0.77	(0.48–1.25)	0.293
Weighted median	7	0.86	(0.52–1.43)	0.564
MR Egger	7	23.42	(0.01–113,590)	0.466
MR PRESSO	7	0.77	(0.48–1.25)	0.334
Myocardial infarction				
Inverse variance weighted	7	0.75	(0.48–1.16)	0.188
Weighted median	7	0.60	(0.35–1.02)	0.057
MR Egger	7	22.35	(0.01–49,622)	0.429
MR PRESSO	7	0.75	(0.48–1.16)	0.236
Ischemic stroke				
Inverse variance weighted	7	0.77	(0.52–1.14)	0.192
Weighted median	7	0.83	(0.52–1.32)	0.437
MR Egger	7	0.34	(0–559.72)	0.773
MR PRESSO	7	0.77	(0.52–1.14)	0.240

*MR PRESSO* MR Pleiotropy RESidual Sum and Outlier, *CI* confidence interval

^a^OR (odds ratio) for engaging in vigorous physical activity (≥ 425 milli-gravities)

**Table 5:** Mendelian randomization estimates between sedentary behavior identified by Doherty et al. [22] in relation to coronary artery disease, myocardial infarction, and ischemic stroke

Method	N SNPs	OR^a^	95% CI	*P* value
Coronary artery disease				
Inverse variance weighted	6	0.91	(0.56–1.49)	0.709
Weighted median	6	0.92	(0.63–1.35)	0.662
MR Egger	6	30.61	(2.25–417)	0.010
MR PRESSO	5	1.10	(0.73–1.66)	0.678
Myocardial infarction				
Inverse variance weighted	6	0.96	(0.59–1.56)	0.855
Weighted median	6	1.04	(0.68–1.60)	0.856
MR Egger	6	21.87	(1.07–446)	0.045
MR PRESSO	6	0.96	(0.59–1.56)	0.862
Ischemic stroke				
Inverse variance weighted	6	1.06	(0.75–1.48)	0.748
Weighted median	6	1.11	(0.75–1.66)	0.599
MR Egger	6	2.07	(0.20–21.36)	0.541
MR PRESSO	6	1.06	(0.75–1.48)	0.761

*MR PRESSO* MR Pleiotropy RESidual Sum and Outlier

^a^OR (odds ratio) for displaying sedentary behavior (energy expenditure ≤ 1.5MET/h)

## Discussion

The current two-sample MR study used GWAS data on self-reported PA from 377,234 individuals, data from 91,084 UK Biobank participants with accelerometer-assessed PA as well as 91,105 subjects with information on sedentary behavior. Further, GWAS data from 42,096 coronary artery disease cases, 27,509 myocardial infarction cases and 40,585 ischemic stroke cases were employed. We found little evidence for an association between PA and sedentary behavior with risk of these cardiovascular outcomes. Although we used the largest currently available GWAS for PA, sedentary behavior, coronary artery disease, myocardial infarction and ischemic stroke, our results are in disagreement with the current scientific concensus with regards to the benefits of regular PA [5, 9]. There may be several reasons for these initially unexpected findings which are outlined below.

In line with our findings, Doherty et al. recently performed a one-sample MR analysis to assess potential relations between device measured PA and sedentary behavior with coronary artery disease, stroke, heart failure, blood pressure, hypertension and anthropometric traits (BMI and body fat %) [22]. No associations were found for overall and moderate PA as well as walking with coronary artery disease, myocardial infarction and stroke [22]. Similarly, van Oort et al. [50] also found no significant association between PA and risk for heart failure. In contrast van der Vegte et al. [51] reported that when sedentarism was specifically defined as watching television, a 1.5 h increase in daily leisure television watching increased the risk for coronary artery disease by 44% independent of BMI and education. We used device measured sedentary behavior and found no evidence of such an association. Further, Zhuang et al. [52] found that self-reported vigorous PA was related to a lower risk for coronary artery disease and myocardial infarction. We used self-reported MVPA and accelerometer-based average acceleration and fraction > 425 milli-gravieties and found no association with cardiovascular risk. Our results suggest that previous observational studies may have been biased and that physical (in)activity is not related to cardiovascular disease risk. The differences between our and previous findings may be explained by the definitions and assessments of PA and sedentary behavior.

A possible explanation for our results is the relation between PA and cardiorespiratory fitness (CRF). We have previously shown that leisure time PA and sports are both positively related to CRF and inversely associated with cardiovascular mortality, whereas occupational PA is neither related to CRF nor to cardiovascular mortality [53]. There is currently a lack of information regarding whether the SNPs related to PA and sedentary behavior used in our analyses are associated with CRF. The phenoscanner analysis found that the SNPs identified by Klimentidis et al. [23] and Doherty et al. [22] were not related to CRF assessed during an exercise test. In addition, even though PA increases CRF [54, 55], it only explains 1–36% of the variance in CRF [56, 57], and CRF has a heritability of at least 50% [58]. Further, animal studies that selectively bred rats for high aerobic capacity demonstrated a progressive genomic divergence caused by drift and selection [59]. These genetic markers do not currently contribute to the observed phenotype but may do so in the future under different environmental conditions. Animal experiments also provide important insight into the relation between PA and CRF. Rats bred for high CRF died significantly earlier when provided with a running wheel compared to their sedentary counterparts [60]. In the same study the authors reported that in monozygotic twins levels of PA were not associated with all-cause mortality. The authors concluded that genetic pleiotropy may influence the biased association observed between high baseline CRF and later mortality. Taken together, differential relations of domain-specific PA and sedentary behavior to CRF and the strong genetic background of CRF may partly explain our null findings.

Our analysis has several notable strengths. The two-sample MR analysis enabled us to combine the largest GWAS on coronary artery disease, myocardial infarction and stroke to date with the largest GWAS on self-reported and device measured PA as well as sedentary behavior to increase the precision of genetically estimated PA, to reduce the potential for weak instrument bias, and to increase statistical power. Objectively measured PA is less prone to recall and response bias than self-reported PA [61].

Our study also had certain limitations. A two-sample MR analysis only provides unbiased risk estimates if the risk factor and outcome sample are derived from the same underlying population. The discovery GWAS of PA and sedentary behavior was based on data from UK Biobank participants of European descent aged 40–70 years [23]. The underlying assumption of using non-specific effects is that the genetic effect on PA and sedentary behavior does not change with age. This may be a limitation because the heritability of PA decreases with age [62]. Given the limited age range of UK Biobank participants and the inclusion of individuals from European ancestry, our results may not be generalizable to other age groups or ancestral populations. Hence, our findings need to be replicated in other age groups and populations. Further, one may argue that the dichotomized exposures (i.e. accelerometer fractions > 425 milli-gravities and sedentary behavior) could violate the exclusion restriction (the genetic variant can influence the outcome via the continuous risk factor even if the binary exposure does not change) [63]. However, this is unlikely to be the case as we also used accelerometer-assessed PA as a continous variable. In addition, the UK Biobank PA GWAS [22, 23] included middle-aged to late-middle aged adults and thus identified SNPs associated with PA at that age. However, previous research based on twin correlations suggests that the genetic contribution to this trait varies across the age range [62]. This means that the genetic effects of time-varying exposures could be heterogeneous across age. Unfortunately, there are currently no GWAS data available that identified genetic variants associated with objectively-assessed physical activity at younger ages. Thus, estimates of PA derived from MR reflect long-term PA exposure in adulthood. Hence, we cannot determine whether higher PA in earlier life may have an association with CAD, MI or ischemic stroke. Our analysis assumes a linear relationship between the risk factors and the outcome. Quantitative estimates maybe misleading if the true relationship is non-linear; although estimates are still reflective of the presence and direction of the population-averaged causal effect [64]. While our proportion of explained variance in PA and sedentary behavior by the SNPs is statistically sufficient, the R^2^ are very small and may be considered a limitation.

Cardiovascular disease is the number one cause for morbidity and mortality worldwide [1–3]. PA is recommended in primary and secondary disease prevention settings to reduce the burden of coronary artery disease, myocardial infarction and stroke [4]. Our results suggest that genetically determined self reported and device measured PA as well as sedentary behavior are not related with cardiovascular disease outcomes. Further, results of MR studies do not necessarily inform how a PA intervention at a specific time in life for a predetermined duration (e.g. cardiac rehabilitation) would work for reducing CAD, MI or ischemic stroke. The results of these analysis should not be interpreted as a means to not recommend exercise to patients since exercise improves overall health irrespective of whether or not cardiovascular endpoints are reached [4].

## Supplementary Information

Below is the link to the electronic supplementary material.

Supplementary file1 (DOCX 565 kb)

## Data Availability

Data supporting the findings of this study are available within the paper and its supplementary information files.

## Code availability

The code can be requested from the authors for quality control purposes.

## References

[1] Benjamin EJ (2018). Heart disease and stroke statistics-2018 update: a report from the American Heart Association. Circulation.

[2] Atlas Writing G (2018). European society of cardiology: cardiovascular disease statistics 2017. Eur Heart J.

[3] Wang H (2016). Global, regional, and national life expectancy, all-cause mortality, and cause-specific mortality for 249 causes of death: a systematic analysis for the Global Burden of Disease Study 2015. The Lancet.

[4] Physical Activity Guidelines Advisory Committee, Physical activity guidelines advisory committee scientific report, in Washington, DC: US Department of Health and Human Services (2018)

[5] Kraus WE (2019). Physical activity, all-cause and cardiovascular mortality, and cardiovascular disease. Med Sci Sports Exerc.

[6] Cheng W (2018). Associations of leisure-time physical activity with cardiovascular mortality: a systematic review and meta-analysis of 44 prospective cohort studies. Eur J Prev Cardiol.

[7] Wahid A (2016). Quantifying the association between physical activity and cardiovascular disease and diabetes: a systematic review and meta-analysis. J Am Heart Assoc.

[8] Howard VJ, McDonnell MN (2015). Physical activity in primary stroke prevention. Stroke.

[9] Ding D (2019). Towards better evidence-informed global action: lessons learnt from the Lancet series and recent developments in physical activity and public health. Br J Sports Med.

[10] Powell KE (2015). The scientific foundation for the physical activity guidelines for Americans, 2nd edition. J Phys Act Health.

[11] Warren JM (2010). Assessment of physical activity—a review of methodologies with reference to epidemiological research: a report of the exercise physiology section of the European Association of Cardiovascular Prevention and Rehabilitation. Eur J Cardiovasc Prev Rehabil.

[12] Prince SA (2008). A comparison of direct versus self-report measures for assessing physical activity in adults: a systematic review. Int J Behav Nutr Phys Act.

[13] Diaz KM (2017). Patterns of sedentary behavior and mortality in US middle-aged and older adults: a national cohort study. Ann Intern Med.

[14] Dohrn I-M (2018). Accelerometer-measured sedentary time and physical activity—a 15 year follow-up of mortality in a Swedish population-based cohort. J Sci Med Sport.

[15] LaMonte MJ (2018). Accelerometer-measured physical activity and mortality in women aged 63 to 99. J Am Geriatr Soc.

[16] Lee IM (2017). Accelerometer-measured physical activity and sedentary behavior in relation to all-cause mortality: the Women's Health study. Circulation.

[17] Matthews CE (2016). Accelerometer-measured dose-response for physical activity, sedentary time, and mortality in US adults. Am J Clin Nutr.

[18] Evenson KR, Wen F, Herring AH (2016). Associations of accelerometry-assessed and self-reported physical activity and sedentary behavior with all-cause and cardiovascular mortality among US adults. Am J Epidemiol.

[19] Smith GD (2006). Randomised by (your) god: robust inference from an observational study design. J Epidemiol Community Health.

[20] Hingorani A, Humphries S (2005). Nature's randomised trials. The Lancet.

[21] Burgess S, Foley CN, Zuber V (2018). Inferring causal relationships between risk factors and outcomes from genome-wide association study data. Annu Rev Genomics Hum Genet.

[22] Doherty A (2018). GWAS identifies 14 loci for device-measured physical activity and sleep duration. Nat Commun.

[23] Klimentidis YC (2018). Genome-wide association study of habitual physical activity in over 377,000 UK Biobank participants identifies multiple variants including CADM2 and APOE. Int J Obes (Lond).

[24] Fry A (2017). Comparison of sociodemographic and health-related characteristics of UK biobank participants with those of the general population. Am J Epidemiol.

[25] Guo W, Key TJ, Reeves GK (2019). Accelerometer compared with questionnaire measures of physical activity in relation to body size and composition: a large cross-sectional analysis of UK Biobank. BMJ Open.

[26] Doherty A (2017). Large scale population assessment of physical activity using wrist worn accelerometers: the UK biobank study. PLoS ONE.

[27] Purcell S (2007). PLINK: a tool set for whole-genome association and population-based linkage analyses. Am J Hum Genet.

[28] Nikpay M (2015). A comprehensive 1,000 Genomes-based genome-wide association meta-analysis of coronary artery disease. Nat Genet.

[29] Malik R (2018). Multiancestry genome-wide association study of 520,000 subjects identifies 32 loci associated with stroke and stroke subtypes. Nat Genet.

[30] Deloukas P (2013). Large-scale association analysis identifies new risk loci for coronary artery disease. Nat Genet.

[31] Burgess S (2014). Sample size and power calculations in Mendelian randomization with a single instrumental variable and a binary outcome. Int J Epidemiol.

[32] Burgess S (2019). Guidelines for performing Mendelian randomization investigations. Wellcome Open Res.

[33] Benjamini Y, Hochberg Y (1995). Controlling the false discovery rate: a practical and powerful approach to multiple testing. J R Stat Soc Ser B (Methodol).

[34] Hemani G (2018). The MR-Base platform supports systematic causal inference across the human phenome. Elife.

[35] Team RC (2013) R: a language and environment for statistical computing

[36] Smith GD et al (2019) STROBE-MR: guidelines for strengthening the reporting of Mendelian randomization studies. PeerJ Preprints

[37] Bowden J (2017). A framework for the investigation of pleiotropy in two-sample summary data Mendelian randomization. Stat Med.

[38] Hemani G, Bowden J, Davey-Smith G (2018). Evaluating the potential role of pleiotropy in Mendelian randomization studies. Hum Mol Genet.

[39] Burgess S, Thompson SG (2011). Avoiding bias from weak instruments in Mendelian randomization studies. Int J Epidemiol.

[40] Kamat MA (2019). PhenoScanner V2: an expanded tool for searching human genotype-phenotype associations. Bioinformatics.

[41] Buniello A (2019). The NHGRI-EBI GWAS Catalog of published genome-wide association studies, targeted arrays and summary statistics 2019. Nucleic Acids Res.

[42] Watanabe K (2019). A global overview of pleiotropy and genetic architecture in complex traits. Nat Genet.

[43] Dogra S (2019). Effects of replacing sitting time with physical activity on lung function: an analysis of the Canadian Longitudinal Study on Aging. Health Rep.

[44] Jones PR, Ekelund U (2019). Physical activity in the prevention of weight gain: the impact of measurement and interpretation of associations. Curr Obes Rep.

[45] Nocon M (2008). Association of physical activity with all-cause and cardiovascular mortality: a systematic review and meta-analysis. Eur J Cardiovasc Prev Rehabil.

[46] Sanderson E (2019). An examination of multivariable Mendelian randomization in the single-sample and two-sample summary data settings. Int J Epidemiol.

[47] Pulit SL (2019). Meta-analysis of genome-wide association studies for body fat distribution in 694 649 individuals of European ancestry. Hum Mol Genet.

[48] Lee JJ (2018). Gene discovery and polygenic prediction from a genome-wide association study of educational attainment in 1.1 million individuals. Nat Genet.

[49] Bowden J, Hemani G, Davey-Smith G (2018). Invited Commentary: Detecting Individual And Global Horizontal Pleiotropy In Mendelian Randomization-A Job For The Humble Heterogeneity Statistic?. Am J Epidemiol.

[50] van Oort S (2020). Modifiable lifestyle factors and heart failure: a Mendelian randomization study. Am Heart J.

[51] van de Vegte YJ (2020). Genome-wide association studies and Mendelian randomization analyses for leisure sedentary behaviours. Nat Commun.

[52] Zhuang Z (2020). Association of physical activity, sedentary behaviours and sleep duration with cardiovascular diseases and lipid profiles: a Mendelian randomization analysis. Lipids Health Dis.

[53] Bahls M (2018). Association of domain-specific physical activity and cardiorespiratory fitness with all-cause and cause-specific mortality in two population-based cohort studies. Sci Rep.

[54] Myers J (2015). Physical activity and cardiorespiratory fitness as major markers of cardiovascular risk: their independent and interwoven importance to health status. Prog Cardiovasc Dis.

[55] DeFina LF (2015). Physical activity versus cardiorespiratory fitness: two (partly) distinct components of cardiovascular health?. Prog Cardiovasc Dis.

[56] Williams PT (2001). Physical fitness and activity as separate heart disease risk factors: a meta-analysis. Med Sci Sports Exerc.

[57] Myers J (2004). Fitness versus physical activity patterns in predicting mortality in men. Am J Med.

[58] Bouchard C (1999). Familial aggregation of Vo 2 max response to exercise training: results from the HERITAGE Family Study. J Appl Physiol.

[59] Ren YY (2013). Genetic analysis of a rat model of aerobic capacity and metabolic fitness. PLoS ONE.

[60] Karvinen S (2015). Physical activity in adulthood: genes and mortality. Sci Rep.

[61] Dowd KP (2018). A systematic literature review of reviews on techniques for physical activity measurement in adults: a DEDIPAC study. Int J Behav Nutr Phys Act.

[62] Vink JM (2011). Variance components models for physical activity with age as modifier: a comparative twin study in seven countries. Twin Res Hum Genet.

[63] Burgess S, Labrecque JA (2018). Mendelian randomization with a binary exposure variable: interpretation and presentation of causal estimates. Eur J Epidemiol.

[64] Burgess S, Davies NM, Thompson SG (2014). Instrumental variable analysis with a nonlinear exposure-outcome relationship. Epidemiology.

